# Romosuzumab used to treat a 29-year-old patient with anorexia nervosa related osteoporosis – A case report

**DOI:** 10.1016/j.bonr.2024.101803

**Published:** 2024-09-11

**Authors:** Pashija Demolli, Diana Frey

**Affiliations:** University Hospital Zurich (USZ), Department of Rheumatology, Switzerland

**Keywords:** Osteoporosis, Low bone mineral density, Anorexia nervosa, Eating disorders, Premnopausal osteoporosis, Osteoanabolic treamtment, Osteoporotic fractures

## Abstract

Osteoporosis and decreased bone density is a frequent complication of anorexia nervosa (AN). As of yet, there have been no studies of accomplished treatment of AN-related osteoporosis with romosuzumab, a monoclonal antibody to sclerostin. We report the first case of a premenopausal, 29-year old patient in Switzerland with decreased bone density and osteoporotic fractures due to anorexia nervosa, who completed the treatment with romosuzumab. There was a significant increase in bone mineral density (BMD) after 12 months of therapy. No serious side effects were reported. To date, only bisphosphonates, denosumab and teriparatide have been evaluated in treatment of AN-related osteoporosis in adolescents and premenopausal individuals respectively. Our report demonstrates that romosuzumab might be an alternative treatment option in patients with anorexia nervosa who are at high risk for osteoporotic fractures. To assess the efficacy and safety of romosuzumab in individuals with AN further studies are needed.

## Highlights

• Romosuzumab demonstrates a significant osteoanabolic effect and increases bone mineral density in young anorexia nervosa patients significantly
• Treatment with romosozumab showed superior clinical efficacy to bisphosphonates and teriparatide in terms of BMD of the lumbar spine, femoral neck and total hip
• Romosuzumab treatment for 12 months was well tolerated

## 1 Background

Low bone mineral density is a common and severe medical complication of Anorexia nervosa and can lead to an increased fracture risk (Vestergaard et al., 2002; Frølich et al., 2020; Søeby et al., 2023). Various endocrine manifestations of AN contribute to alterations in bone metabolism. Low body weight and gonadal dysfunction are associated with low leptin and estrogen levels and appear to be the main contributing risk factors. Their effects on bone metabolism have been assessed in several studies (Frølich et al., 2017; Khosla et al., 2012). Additional co-occurring mood disorders such as depression and anxiety and the need for longtime medication with selective serotonin reuptake inhibitors (SSRIs) can reduce bone formation. Other factors that increase the likelihood of developing osteoporosis in AN patients are identical to the risk factors in the general population and include genetic predisposition, low calcium intake, vitamin d deficiency, glucocorticoids and other medication (e.g. anti-epileptic drugs, proton pump inhibitors), nicotine and an inactive lifestyle.

Regarding treatment of AN-associated bone disease, weight gain has a major impact on bone mineral density (Frølich et al., 2017). Since a majority of women with anorexia nervosa experience amenorrhea, restoration of gonadal dysfunction by hormone replacement is another common treatment (Khosla et al., 2012). Furthermore antiresorptive and osteoanabolic therapy with bisphosphonates, denosumab and teriparatide have been reported as treatment of AN-related osteoporosis (Haines et al., 2022; Golden et al., 2005; Miller et al., 2011). So far, romosuzumab, a monoclonal antibody to sclerostin, has not been assessed in the treatment of AN-related osteoporosis in premenopausal women.

### 1.1 The role of Sclerostin

Sclerostin is a signaling molecule, synthesized by osteocytes. It is a potent inhibitor of bone formation. Sclerostin binds low-density lipoprotein receptor-related protein (LRP 4/5/6) on the surface of osteoblasts, causing an inhibition of the Wnt signaling pathway. Thus, osteoblast differentiation and/or function is suppressed, and bone formation blocked. High Sclerostin levels also cause bone resorption by increasing RANKL expression. RANK Ligand is a signaling molecule, which regulates osteoclast activity. It promotes differentiation, activation, and survival of osteoclasts via interaction with its receptor RANK, thus activating bone resorption. Evaluation of Sclerostin levels in anorexia nervosa has shown increased sclerostin levels in AN patients (Maïmoun et al., 2014), suggesting that Sclerostin levels may play a key effect in bone loss in AN individuals. Also, the study of Faje AT, Fazeli PK, Katzman DK, et al. (Faje et al., 2012) hints, that transdermal estradiol replacement in AN adolescents does not change sclerostin levels whereas healthy controls who receive estrogen replacement show decreased sclerostin levels.

Therefore, treatment with romosuzumab, targeting sclerostin and decreasing its level has the potential to increase bone formation in this group of patients.

## 2 Case presentation

A 29-year-old female diagnosed with Anorexia nervosa (AN) at the age of 16 years and history of decreased bone mineral density presented to our clinic in 2014 for a follow-up. She was diagnosed with low bone mass in the year of 2014 (T-Score lumbar spine: −2.4, T-Score total hip: −2.6, *Z*-Score lumbar spine: −2.5, Z-Score total hip: −2.6). Besides AN, there were additional risk factors contributing to higher risk for osteoporotic fractures; a secondary amenorrhea since 2013 and positive family history for osteoporosis (mother with osteoporosis). No fractures had occurred in the previous history. A hormone replacement treatment was started in the year of 2020. After the diagnosis in 2014, she participated in a clinical trial and completed 2 years of therapy with teriparatide. After two years of teriparatide treatment there was a significant increase in lumbar spine bone density, but no improvement for the total hip area and femoral neck (Table 2). Subsequently she was treated with intravenous ibandronate for 6 years (2016–2019). Follow up osteodensitometries in 2016 and 2019 showed a further significant increase in bone mineral density of the lumbar spine (Table 2). During this period, she suffered no bone fractures. Relapses of AN-related loss of body weight occurred several times, with the lowest BMI being 12.1 Kg/m2 (2015) and the highest 17.5 Kg/m2 (2019). On routine surveillance examination in the year of 2022 the patient reported a non-traumatic rib fracture she had suffered in the previous year and a body weight loss of 10 Kg (to body weight of 42 Kg / BMI 13.6 Kg/m2) since the previous examination.

Clinical findings and dual-energy x-ray absorptiometry (DXA) showed a severely decreased BMI of 13.6 Kg/m2 (2019: 17.1 Kg/m2) and a significantly lowered bone mineral density in the lumbar spine, mean total hip and femoral neck, with a significant decline in the hip total area compared to the previous DXA of 2019. Other, secondary causes (Table 1a) of the low bone density and the additional loss were investigated with clinical and laboratory examination. The laboratory results showed a decreased P1NP level, suggesting an insufficient bone formation. There were no other relevant findings (Table 1b).

**Table 1a:** Secondary causes of osteoporosis.

Endocrine disease	Hematological disease	Gastrointestinal disease	Rheumatological disease	Congenital disease	Medications	others
Diabetes mellitus Hyperparathyreoidism Hypogonadism Hyperthyroidism Hyperprolactinemia	Multiple myeloma Thalassemia Heamochromatosis Mastocytosis	Chronic inflammatory bowel disease Malabsorption and Malnutrition Primary biliary cirrhosis Chronic liver disease Lactose intolerance	Rheumatoid arthritis Ankylosing spondylitis Other rheumatological conditions	Osteogenesis imperfecta Ehlers-Danlos syndrome Marfan's syndrome	E.g. antiepileptics Synthetic Glucocorticoids (e.g. prednisone) aromatase inhibitors GnRH agonists Glitazones Depo-Provera Tamoxifen (in premenopausal women) Selective serotonin reuptake inhibitors (SSRIs) Pantoprazole	Neurological disorders Organ transplantation Anorexia nervosa Severe chronic renal insufficiency Idiopathic hypercalciuria Chronic alcoholism Smoking HIV Immobilization

**Table 1b:** Secondary causes of osteoporosis; laboratory tests.

Laboratory parameter	Role/underlying conditions	Findings in our patient (reference values)
Laboratory testing for osteoporosis		
Differentiated blood count	Hematologic pathology	Normal findings
Erythrocyte sedimentation rate	Increased in multiple myeloma	–
Serum calcium (albumin corrected or ionized)	Elevated in primary hyperparathyroidism or skeletal metastases Decreased in secondary hyperparathyroidism, malabsorption	1.24 mmol/l (1.15–1.33, ionized)
Serum phosphate	Decreased in secondary hyperparathyroidism, malabsorption, iron infusions	1.27 mmol/l (0.87–1.45)
Sodium/potassium	Hyponatremia/Hypokalemia	140 mmol/l (136–145) 4.1 mmol/l (3.5–5.1)
Protein	Hyper- or hypoproteinemia of various causes	68 g/l (66–87)
Albumin	Protein deficiency, correction factor for calcium	48 g/l (40–49)
Alkaline phosphatase (AP)	Elevated in osteomalacia, fractures Decreased in hypophosphatasia	43 U/l (35–105)
Gamma-GT	If alkaline phosphatase increased to differentiate from hepatic alkaline phosphatase elevation	16 U/l (<40)
Serum creatinine incl. Glomerular filtration rate	Renal osteopathy - Renal function before therapy with bisphosphonates	70 umol/l (44–80) 101 ml/min
Serum protein electrophoresis or immunofixation	Pathological in gammopathy	–
TSH (possibly fT3 and fT4)	Decreased in hyperthyroidism or iatrogenic under-medication Caution: latent hyperthyroidism can also have a negative effect on bone metabolism	0.89 mU/l (0.16–4.25)
25-OH-vitamin D	Decreased in case of insufficient intake, malabsorption - Optimal levels are 30-60 g/L (resp. 75-150 nmol/L)	100.0 μg/l (>20.0)

Additional examinations according to medical history, clinical findings, or in the case of severe and/or unclear osteoporosis		
Intact PTH	To differentiate calcium changes, primary/secondary hyperparathyroidism, tumor hypercalcemia, and normocalcemic hyperparathyroidism	24.4 ng/l (15–65)
Tryptase	To check for mastocytosis	–
Anti-transglutaminase antibodies and anti-gliadin antibodies	For screening for celiac disease, possibly including IgA levels	–
Prolactin	Including screening for macroprolactin and prolactinoma	–
Total testosterone	For screening for male hypogonadism	–
1–25-Di-OH-Vitamin D	To check for hydroxylation disorders of the kidney	–
Basal cortisol	Including midnight salivary cortisol or cortisol in 24-h urine, for screening for Cushing's disease	400 nmol/l (133–537)
Ferritin, transferrin, and transferrin saturation levels	To screen for hemochromatosis	41 μg/l (13–150)
CDT	If there is alcohol abuse	–
Beta-crosslaps and possibly P1NP	For evaluating bone remodeling rate	Beta-Crosslaps: 0.25 ng/ml (<0.57, premenopausal) P1NP: 10.4 ng/ml (15.1–58.6, premenopausal)
HIV	Screening	–
Hepatitis B and C	Screening	–

Due to the rib fracture without a previous trauma whilst continuing regular treatment with intravenous ibandronate, the indication for osteoanabolic treatment was given. Since there was no contraindication, we discussed to start therapy with romosuzumab, being the only alternative option to teriparatide for anabolic therapy. After discussion of potential risks and side effects, the patient agreed to start therapy with monthly romosuzumab injections. In addition to the dual-energy x-ray absorptiometry (DXA) we performed a laboratory test prior to the treatment to assess the baseline values of the bone turnover markers (P1NP and beta-crosslaps/beta-CTX) and continued evaluation during treatment for monitoring the therapeutic success. Furthermore we checked the blood Calcium level since it can be lowered due to treatment with romosuzumab (potential side effect). During the treatment period clinical and laboratory follow-ups were performed after 1, 3 and 12 months. After completing the treatment bone mineral density was measured.

## 3 Outcome and follow-up

The patient completed a 12-month treatment with romosuzumab. Because of the low body weight (BMI: 13.6 Kg/m2) we decided to start treatment with only half of the normal dose of romosuzumab (105 mg per month) for 3 months. During this time the patient reported mild muscle and joint pain of the arms and legs, occurring within the first day of the injections and lasting for another 1 to 2 days. The symptoms would resolve without treatment. During the following months (4, 5 and 6) of treatment, the patient received the full dose of romosuzumab (210 mg per month). The patient reported an increase of side effects with the full dose, with significantly more muscle and joint pain, additionally weakness in the legs and brittleness of the nails. Due to persisting side effects, we decided to continue treatment with the reduced dose of 105 mg. The new side effects would resolve immediately after reducing the dose, whereas the mild muscle and joint pain would persist during the whole treatment. No other side effects were reported.

Laboratory follow-ups in the first 3 months of treatment showed a significant increase of the bone formation marker P1NP, gradually decreasing to nearly baseline value by month 12. The bone resorption marker beta-CTX decreased markedly below baseline after 3 months, remaining low after 12 months of treatment (Table 2).

**Table 2:** Laboratory tests of the patient before, during and after therapy with Romosuzumab.

	Jun 22	Jul 22	Sep 22	Mai 23
	Before treatment	1 mo	3 mo*	12 mo
Laboratory parameter				
β-CTX (ng/ml)	0.25	0.2	<0.03	0.12
P1NP (ng/ml)	10.4	54.3	67.7	11.7
Calcium (mmol/l)	1.24	1.22	1.24	1.19

Dual-energy x-ray absorptiometry (DXA) performed after completion of treatment of 12-month romosuzumab showed a significant increase in bone mineral density with a BMD change of +17.4 % for the lumbar spine (L1-L4), +4.8 % for the total hip and + 6.2 % for the femoral neck (Table 3). Treatment is currently continued with Zoledronic acid (5 mg, intravenous) once a year, next DXA planned in May 2025.

**Table 3:** Bone mineral density (BMD g/cm2 and Z scores) of the lumbar spine, femoral neck and total hip before treatment, after Teriparatide, after Ibandronat and after 12 months of treatment with Romosuzumab (BMD change in % to previous measurement).

Year of examination and BMI (Kg/m2)	2014 13.4 Kg/m2	2015 12.1 Kg/m2	2016 14.8 Kg/m2	2019 17.1 Kg/m2	2022 13.6 Kg/m2	2023 14.5 Kg/m2
	Before treatment	After teriparatid (12 months)	After teriparatid (24 months)	After ibandronate (3 years)	After ibandronate (6 years) and before Romosuzumab treatment respectively	After completed treatment with romosuzumab (12 months^a^)
Lumbar spine (L1-L4)						
Z score	−2.5	−2.6	−2.4	−2.2	−2.4	−1.1
BMD, g/cm2	0.753	0.746 (−0.9 %)	0.766 (+2.7 %)	0.796 (+2.6 %)	0.787 (−1.1 %)	0.924 (+17.4 %)

Femoral neck						
Z score	−1.8	−1.8	−2.3	−2.3	−2.2	−1.9
BMD, g/cm2	0.646	0.650 (+0.6 %)	0.590 (−10.2 %)	0.592 (+0.3 %)	0.594 (+0.3 %)	0.631 (+6.2 %)

Total hip						
Z score	−2.6	−2.6	−2.7	−2.7	−2.9	−2.6
BMD, g/cm2	0.625	0.626 (+0.2 %)	0.608 (−2.8 %)	0.608 (+0.1 %)	0.590 (−3.1 %)	0.618 (+4.8 %)

a During 9 months half-dose Romosuzumab treatment due to side effects.

## 4 Discussion

Anorexia nervosa is a psychiatric disorder characterized by a severe restriction of food intake, significant weight loss, and morbid fear of gaining weight (Zipfel et al., 2015). It triggers numerous endocrinological changes that are directly related to the degree of malnutrition. The hypothalamus, pituitary gland, gonads, adrenal gland, and the adipokines leptine are all affected, leading to an alteration in a wide range of hormones and signaling peptides. One of the most concerning comorbidities of anorexia nervosa is the reduction in bone density, which not only leads to the development of osteoporosis but also increases the risk of fractures not only during the time of AN, but also later in life, thus contributing to higher morbidity, lower quality of life and higher health care costs. Cortical and trabecular bone are both equally affected and may therefore be prone to osteoporotic fractures. Additional risk factors such as a genetic predisposition contribute to the development of osteoporosis and increase the risk for fractures. A recent study has found that AN is associated with a 46 % higher risk of any sort of fractures up to 40 years after diagnosis (Søeby et al., 2023).

The primary treatment goals for AN-related osteoporosis are prevention of vertebral and non- vertebral fractures and an increase in the patient's bone mineral density. Restoration of body weight and the resumption of the menstrual cycle are known to have a strong influence on bone mineral density (Frølich et al., 2017; Khosla et al., 2012). A conservative management includes supplementation with vitamin D und Calcium. Additionally an hormone replacement therapy is established, since AN-patients very often develop secondary amenorrhea. Antiresorptive therapy is used in more severe cases, especially if osteoporotic fractures are present. However, there is not much data regarding antiresorptives in young, premenopausal patients. So far Bisphosphonates (Golden et al., 2005; Miller et al., 2011) and Denosumab (Haines et al., 2022) have been assessed in AN. Alendronate showed a slight increase of BMD in the lumbar spine and femoral neck (3.5 % and 4.4 % respectively) (Golden et al., 2005), whereas Risedronate and Denosumab led to a slight increase of the lumbar spine BMD only (Haines et al., 2022). A pilot study with Teriparatide (TPT) in young AN-patients showed significant increase in BMD (13.5 % in the lumbar spine, 5.0 % in the femoral neck and 4.0 % in the total hip area) (Milos et al., 2021) after 24 months treatment. Further, a randomized controlled trial has shown that Teriparatide administration increases spine BMD substantially after only 6 months of therapy in women with AN (Fazeli et al., 2014). There are no larger studies with TPT in young patients. As for Romosuzumab, so far there have been no reports of a treatment in young patients with anorexia nervosa.

Our patient had a long history of anorexia nervosa, with a dramatically low BMI of 13.4 Kg/m2, secondary amenorrhea, low calcium intake, vitamin d deficiency, and a potential genetic predisposition for osteoporosis with her mother being diagnosed with postmenopausal osteoporosis. There was no history of alcohol and nicotine consumption nor other health issues or medication affecting bone health. Besides regular calcium and vitamin d supplementation, hormone replacement treatment with a biphasic hormone combination (estrogen/gestagen) was established in the year of 2013. Treatment with teriparatide showed a minimal improvement of bone mineral density in the lumbar spine (+2.7 % after 24 months). There was a decrease in bone mineral density for the total hip (−2.8 %) and femoral neck area (−10.2 %), as shown in Table 1a, Table 1b. A further weight loss in the year of 2015 could be a reason for this significant bone mineral density loss (BMI 12.1 Kg/m2) despite the osteoanabolic treatment with teriparatide. Another reason might be the known increase of cortical porosity under teriparatide treatment, leading to apparent adverse changes in areal bone density in cortical regions such as the hip. This could explain the observed decrease in bone mass in the hip area when assessed using DXA. However, after antiresorptive treatment an increase of BMD of cortical bone is usually seen in postmenopausal non-AN-patients.

**Figs. 1 & 2:**
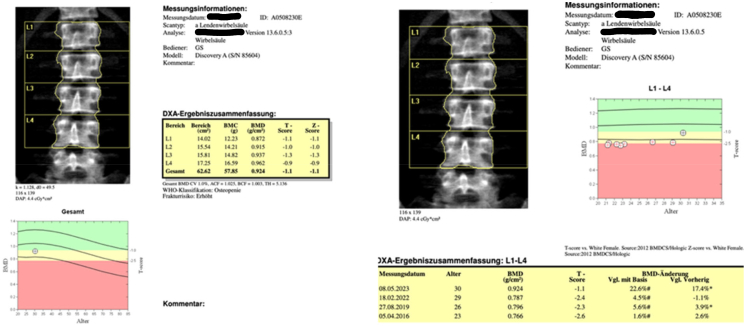
DXA reports for lumbar spine after completing treatment with romosuzumab.

A further improvement of the lumbar spine BMD of +2.6 % was observed after a subsequent 3-year treatment with intravenous ibandronate. However, there was also a significant weight gain and change in BMI from 13.4 kg/m2 to 17.1 kg/m2 during this period – being another potential factor of bone density increase. Neither teriparatide nor ibandronate led to an increase in BMD of the total hip and femoral neck area. Compared to the first osteodensitometry there was even a loss of BMD for the hip and femoral neck during these treatments (Table 1a, Table 1b). In addition, during therapy with ibandronate our patient suffered a rib fracture without a previous trauma. The most significant positive change was observed after 12 months of treatment with romosuzumab. The lumbar spine BMD value increased by 17.4 % leading to a normal bone density value for the first time in this patient (Figs. 1 & 2).

**Figs. 3 & 4:**
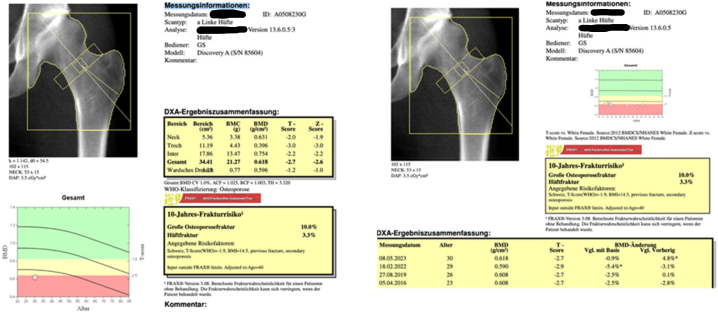
DXA reports for total hip and femoral neck after completing treatment with romosuzumab.

The femoral neck BMD improved by 6.8 %, thus returning to to the base line value in the lower normal range. The total hip BMD increased as well (+4.8 %), for the first time since measuring reaching the base line value before any treatment (Figs. 3 and 4). Notably there was only a minimal weight gain and BMI change during the treatment with romosuzumab (BMI 13.6 Kg/m2 before and 14.5 Kg/m2 after treatment). Therefore, we can assume, that the significant improvement in BMD was not promoted by weight gain. There was no change in risk constellation during this period. Aside from transient joint and muscle pain our patient experienced no major adverse side effects from the therapy with romosuzumab.

## 5 Conclusion

Based on our findings, 12 months of romosozumab treatment led to a significant increase in BMD values in the lumbar spine, femoral neck, and total hip of our AN-patient, without any subsequent fractures and adverse effects. In conclusion, this case report provides evidence that romosuzumab may be an effective treatment for AN-related osteoporosis in premenopausal individuals, as it has the potential to improve BMD and reduce the risk of fractures. However, further research is needed to assess the efficacy and safety of this treatment option in a larger population of young AN patients.

## Informed consent statement

Written informed consent was obtained from the patient for the publication of this paper.

## CRediT authorship contribution statement

**Pashija Demolli:** Writing – original draft. **Diana Frey:** Supervision.

## Declaration of competing interest

The authors certify that they have NO affiliations with or involvement in any organization or entity with any financial interest (such as honoraria; educational grants; participation in speakers’ bureaus; membership, employment, consultancies, stock ownership, or other equity interest; and expert testimony or patent-licensing arrangements), or non-financial interest (such as personal or professional relationships, affiliations, knowledge or beliefs) in the subject matter or materials discussed in this manuscript.

## Data Availability

The data that has been used is confidential.
